# The Effect of a High-Frequency Exercise Program During the Transition Period in Young Football Players

**DOI:** 10.3390/sports13090297

**Published:** 2025-09-01

**Authors:** Yiannis Michailidis, Andreas Stafylidis, Athanasios Mandroukas, Eleni Semaltianou, Georgios Karamousalidis, Georgios Antoniou, Vasileios Leftheroudis, Vasilios Mittas, Thomas I. Metaxas

**Affiliations:** Laboratory of Evaluation of Human Biological Performance, Department of Physical Education and Sports Sciences, Aristotle University of Thessaloniki, University Campus of Thermi, 57001 Thessaloniki, Greece; astafylidis@phed.auth.gr (A.S.); amandrou@phed.auth.gr (A.M.); esemalt@phed.auth.gr (E.S.); gkaramou@phed.auth.gr (G.K.); giorgosanton@gmail.com (G.A.); b.leftheroudis@hotmail.com (V.L.); mittasv@hotmail.com (V.M.); tommet@phed.auth.gr (T.I.M.)

**Keywords:** football, strength, aerobic capacity, VO_2_ max, power, jumping ability, body weight, body fat

## Abstract

The transition period in football can negatively affect players’ fitness indicators. However, if appropriate training programs are implemented during this period, these effects can be reversed. The purpose of this study was to investigate the effect of a high-frequency exercise program during the transition period on aerobic capacity, isokinetic torque of the lower limbs, jumping ability, and body composition. The transition period was divided into two phases: the first phase lasted two weeks and involved complete rest from exercise, and the second phase lasted four weeks during which the players completed three aerobic-focused training sessions and two maximum strength training sessions per week. A total of 13 young football players (age 17.8 ± 0.7 years, height 1.78 ± 0.07 m, weight 70.3 ± 8.4 kg) participated in the study. A paired samples *t*-test was applied, and statistical significance was set at *p* < 0.05. The results showed that players improved their maximal oxygen uptake (VO_2_ max) after the program (*p* = 0.037, t = −2.348). The performance in countermovement jump performance and in the isokinetic torque of the right quadriceps showed a decline (*p* = 0.009, t = 3.112 & *p* = 0.004, t = 2.299, respectively), while no changes were observed in any other parameter (*p* > 0.05). The findings suggest that a program with these characteristics can counteract the negative effects typically observed during the transition period. Moreover, with specialized stimuli, improvement may also be observed during the transitional period.

## 1. Introduction

Football is one of the most popular sports worldwide, with millions of male and female athletes participating [1,2]. Performance in football depends on technical and tactical skills, physical fitness, and the psychophysiological characteristics of the players [3]. Advances in sports science have significantly contributed to the development of football at all levels (e.g., video analysis, workload monitoring using global positioning systems) [4,5,6].

In recent decades, football has evolved into a highly demanding sport. In most national leagues, players participate in over 50 matches per year, and teams competing in international tournaments (e.g., Champions League, Europa League, Conference League) may exceed 60 official games [7]. Another characteristic of the sport is the long competitive season, often lasting more than 10 months each year [8]. To meet these demands, a team must significantly develop and maintain all key performance factors throughout most of the year. The annual training cycle is typically divided into three main periods: the preparatory period, the competitive period, and the transition period [9]. Each of these periods has a different duration: the preparatory period lasts approximately 6 weeks, the competitive period around 40 weeks, and the transition period about 6 weeks [9].

As previously mentioned, physical fitness is a crucial determinant of performance in modern football. Today’s footballers demonstrate characteristics of multi-athletes, as high levels of aerobic capacity, strength, speed, and agility are required to meet the demands of the game [3]. For this reason, coaching staff focus on developing these qualities during the annual training cycle, particularly in the preparatory period, and strive to maintain them throughout the season. However, the transition period is often characterized by reduced training frequency or even complete cessation of training for several days. This pause is necessary for the physical, mental, and emotional recovery of the players. Nevertheless, changes in training load during this period can negatively impact players’ physical abilities. Specifically, a reduction in the number of sessions, training volume, and intensity can adversely affect both aerobic and anaerobic capacity [10,11,12]. As a result, more training is required during the preparatory period to restore fitness levels.

On the other hand, the transition period, being free of competitive obligations, may be ideal for addressing individual weaknesses [9,11]. This is particularly important for youth players, as they have significant potential for improvement in all areas that influence football performance. Therefore, after ensuring adequate physical and psychological recovery at the end of the competitive season (typically 15–20 days of rest), players may follow an individualized training program aimed at maintaining and gradually improving physical fitness. This approach can reduce the time needed during preseason to reach optimal fitness levels [13].

Thus, the aim of this study is to investigate the effect of a six-week transitional training program on body composition, aerobic capacity, jumping ability, and strength in young football players. We hypothesize that the transitional training program will restore players’ physical condition to pre-transition levels.

## 2. Materials and Methods

### 2.1. Study Design

After the completion of their competitive obligations, the football players continued training with their team for an additional month. During the final week before the start of the transition period, they visited the laboratory for assessments of anthropometric characteristics, aerobic capacity, jumping ability, and isokinetic torque of the lower limbs. This was followed by a two-week vacation during which the players did not engage in any exercise. Then, over the next four weeks, they followed the transitional training program. Two to three days after the program’s completion, they returned to the laboratory for reassessment prior to the beginning of the preseason.

### 2.2. Participants

The study included 13 young soccer players who competed in their country’s U17 national league (chronological age: 17.8 ± 0.7 years, CI: 17.4–18.3; training age: 11.3 ± 1.8 years, CI: 10.3–12.4). The football players, according to a classification proposed in the literature [14], belong to category 3–4 (highly trained/national level–elite/international level), since a significant percentage of the participants are members of their age-category national team, and, furthermore, the team takes part each year in at least two international tournaments. Player characteristics are presented in Table 1. Two players were excluded from the study for not meeting the following criteria: (a) no injury for at least one month prior to the initial assessment or during the transition period, (b) no use of medication, (c) completion of at least 90% of the individual training sessions, and (d) no participation in other sports activities during the training program.

All participants and their legal guardians were informed about the study procedures and provided written informed consent. The study was conducted in accordance with the Declaration of Helsinki and was approved by the institutional review board.

### 2.3. Anthropometric Measurements

Body weight was measured using a TANITA DC-360 digital scale (Tanita Corporation, Tokyo, Japan) with a precision of 0.1 kg. The same device was used to measure body fat percentage, based on the principle of bioelectrical impedance analysis. Standing height was measured using a Seca 220e stadiometer (Seca, Hamburg, Germany) with an accuracy of 0.1 cm.

### 2.4. Isokinetic Strength Testing

The strength of the hamstrings and quadriceps was assessed under isokinetic conditions using a Cybex II dynamometer (Lumex Inc., Ronkonkoma, NY, USA). For each test velocity, the highest torque output was recorded, with corrections made to eliminate the effects of limb mass and machine resistance. Participants were seated in an adjustable chair with straps across the torso, hips, and thighs to prevent extraneous movement. The test leg was positioned at 90° of knee flexion (0° = full extension), aligning the knee joint axis with the dynamometer’s lever arm at the distal lateral femoral condyle. Lever length and resistance pad placement were individually adjusted, with the pad placed just above the medial malleolus. The opposite leg remained relaxed and unsupported.

The movement sequence was fixed: extensions started from 90° flexion and flexions from full extension. Players were instructed to exert maximal effort throughout the full range of motion as quickly and forcefully as possible while keeping their arms crossed. Three repetitions were performed at each speed, and the peak torque value was used for analysis. A 30-s rest was given between reps, and one minute was given between velocity changes. Verbal encouragement was provided to ensure maximum effort. Measurements were taken at 60°, 180°, and 300°/s. The peak isokinetic torque was defined as the highest force value across the full movement. The hamstring-to-quadriceps (H:Q) ratio was calculated by dividing the peak concentric torque of the hamstrings by that of the quadriceps at each speed [15].

### 2.5. Laboratory VO_2_ max Measurement

Maximal oxygen uptake (VO_2_ max) was measured on a motorized treadmill (Pulsar; h/p/Cosmos, Nussdorf-Traunstein, Germany) using a continuous incremental protocol. The initial speed and grade were set at 8 km/h and 0%, respectively. Every two minutes, the speed increased by 2 km/h up to a speed of 12 km/h. Then, from a speed of 14 km/h, the gradient was set at 2%, and the speed increased every 1 min until the subject reached volitional exhaustion.

Oxygen uptake and respiratory indices were measured using a breath-by-breath automated ergospirometry system (Quark CPET, COSMED, Rome, Italy). Prior to testing, the gas analyzer was calibrated using a 2.0 L calibration syringe and certified reference gases. VO_2_ max was defined as the highest value recorded over at least five consecutive breaths (steady-state). Heart rate was also recorded using a Polar Team Pro monitor (10 Hz, Kempele, Finland).

The VO_2_ max measurement was considered valid if 3 out of the following 5 criteria were met: (a) heart rate during the final minute exceeded 95% of the predicted maximum (220 − age), (b) VO_2_ plateaued despite increasing speed (ΔVO_2_ < 150 mL), (c) respiratory exchange ratio (RER = VCO_2_/VO_2_) ≥ 1.1, (d) the participant voluntarily terminated the test despite verbal encouragement, and (e) a high rating of perceived exertion (RPE) on the Borg scale (>17) [16,17].

### 2.6. Countermovement Jump (CMJ) and Squat Jump (SJ)

Jumping ability was assessed using Countermovement Jump (CMJ) and Squat Jump (SJ)**.** Each jump was performed twice, and the best result was used for analysis. For the CMJ, players started from a standing position with hands on hips, performed a self-selected depth countermovement, and then executed a maximal vertical jump. For the SJ, participants began in a static 90° squat position, jumped vertically without any preliminary movement, and aimed to jump as high as possible.

Incorrect attempts were repeated after a one-minute rest. Recovery between attempts was 30 s and three minutes between jump types. All jumps were assessed using the OptoJump optical measurement system (Microgate, Bolzano, Italy), which is known for its strong concurrent validity and excellent test–retest reliability in estimating vertical jump height [18].

### 2.7. Transitional Phase Training Program

**Figure 1:**
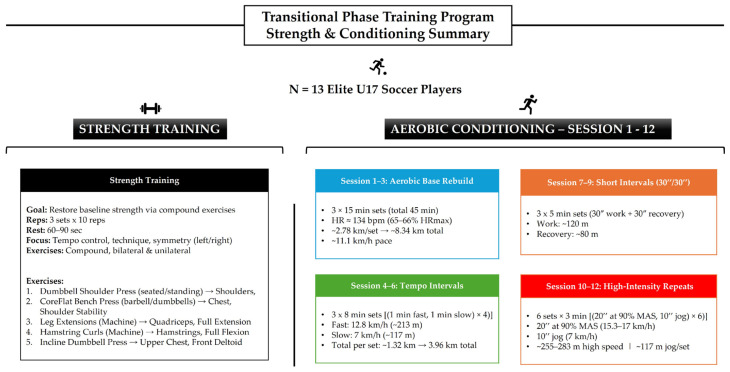
Overview of the transitional phase training program incorporating strength and aerobic conditioning sessions.

The program implemented by the soccer players focused on aerobic capacity and strength. Each week, the players performed five training sessions: aerobic training on Monday, Wednesday, and Friday, and strength training on Tuesday and Thursday. For aerobic capacity, the continuous method was initially used, followed by long interval high-intensity training and short interval high-intensity training. For strength training, the players followed the same program throughout the four weeks (8 sessions), which included 3 sets of 10 repetitions (10 repetition maximum). The program and its contents are presented in Figure 1.

### 2.8. Exercise Protocol

The primary aim of the training program was, as previously noted, to mitigate the effects of detraining. An additional objective was to ensure that the duration of each training session did not exceed one hour. Accordingly, the aerobic conditioning sessions lasted between 40 and 60 min, while the strength training sessions lasted approximately 45 min. Prior to the aerobic sessions, a 15-min warm-up was performed, consisting of running, movement drills, and stretching exercises. Similarly, before strength training sessions, participants completed a 15-min warm-up that included cycling, stretching, and practice sets of the prescribed strength exercises at 50% of their one-repetition maximum (1 RM), which was determined by maximal testing prior to the transition period. The program targeted four major muscle groups: the deltoids, the pectoralis major, and the knee flexors and extensors. Exercise intensity was prescribed at 10 RM.

Note: This transitional program integrates structured strength and aerobic components over 12 sessions to optimize recovery, rebuild submaximal capacity, and restore neuromuscular readiness. Strength training emphasizes bilateral compound movements with optional unilateral modifications. Aerobic training progresses from low-intensity continuous to high-intensity intermittent formats based on velocity and HRmax zones, allowing individualized adaptation. Each aerobic session builds in volume and intensity to facilitate return-to-play conditioning without overloading the system prematurely.

### 2.9. Statistical Analysis

Statistical analysis. A priori power analysis was conducted using G*Power (version 3.1) [19,20] to determine the minimum sample size required for detecting a significant within-subject effect using a two-tailed paired-samples *t*-test. The parameters included a large effect size (ES = 0.80), α error probability of 0.05, and desired statistical power of 0.80. The analysis indicated that a minimum of 12 participants would be required to detect a statistically significant difference. The final sample included 13 participants, thereby exceeding the minimum requirement for adequate statistical power. All statistical analyses were conducted using JASP (version 0.19.3), Jamovi (version 2.6), and SPSS (version 29) [21,22,23]. Descriptive statistics (mean ± standard deviation and 95% CI) were calculated for all outcome variables. Normality of the data distribution was assessed using both the Shapiro–Wilk test and visual inspection of histograms and Q–Q plots. Subsequently, paired-samples *t*-tests were conducted to examine within-subject differences. Effect sizes were calculated using Cohen’s d [24], which was interpreted as small (d < 0.50), medium (d ≥ 0.50 and < 0.80), and large (d ≥ 0.80). Statistical significance was set at *p* < 0.05.

## 3. Results

Descriptive statistics for basic participant characteristics at pre- and post-intervention are presented in Table 1. Paired-samples *t*-tests were conducted to examine within-subject differences across the two time points (Table 1). Chronological age and training experience exhibited minor yet expected increases between pre- and post-intervention. Body weight remained stable over the intervention period, with identical means recorded pre- and post-intervention. Similarly, Body Mass Index (BMI) showed a minimal, non-significant decrease, suggesting that overall mass remained consistent during the off-season phase. Measures of body fat percentage and fat mass (kg) remained unchanged, reflecting no substantial increase. Additionally, muscle mass was preserved, with only a small non-significant decrease, indicating that the implemented off-season training protocol was effective in maintaining lean body mass (Table 1).

Descriptive statistics and paired-samples *t*-test results for cardiovascular and cardiorespiratory variables are presented in Table 2. Among all parameters assessed, statistically significant improvements were observed in maximal oxygen uptake (VO_2_ max), both in relative (mL/kg/min) and absolute terms (mL/min). Specifically, VO_2_ max (mL/kg/min) significantly increased from 55.4 ± 5.1 to 57.1 ± 4.6, while absolute VO_2_ max rose from 3871 ± 539 to 4022 ± 407. Both effect sizes were moderate and suggest meaningful enhancements in aerobic capacity following the intervention period.

Although the remaining variables did not demonstrate statistically significant changes (*p* > 0.05), several parameters showed descriptively higher values based on their means. For instance, anaerobic threshold time increased non-significantly by approximately 7 s. Additionally, maximal exercise time, maximal velocity, and respiratory exchange ratio (RER) showed non-significant post-intervention increases, which may reflect better physiological tolerance at higher workloads. Resting heart rate, systolic, and diastolic pressures remained relatively stable. While not statistically significant, these improvements collectively may suggest that the intervention helped maintain or improve cardiovascular and metabolic efficiency during a period typically associated with detraining in soccer players.

Descriptive statistics and the results of the paired-samples *t*-tests for isokinetic strength and jump performance are presented in Table 3. A significant decrease was observed in right knee extensor strength at 60°/s from pre- to post-intervention. Furthermore, countermovement jump (CMJ) performance also showed a significant decline following the intervention. No other statistically significant changes were observed in knee flexor or extensor torque at any angular velocity, with all corresponding *p*-values > 0.05. Despite this fact, several variables exhibited modest improvements in mean values post-intervention. These include right knee extensor torque at 180°/s and 300°/s, and right knee flexor torque at 180°/s and 300°/s, all of which demonstrated non-significant increases in mean performance compared to pre-intervention, although none reached significance (*p* > 0.05). Similarly, left knee extensor torque at 60°/s and 180°/s and left knee flexor torque at 60°/s, 180°/s, and 300°/s also exhibited minor increases or remained relatively stable. These findings suggest that the off-season intervention program may have contributed to maintaining or non-significantly improving neuromuscular performance in specific parameters, despite the absence of statistical significance.

## 4. Discussion

The results of the present study indicated that the implementation of the transitional training program led to an improvement in the players’ aerobic capacity, while almost all of the strength variables were maintained compared to the pre-transition period. Therefore, the initial hypotheses were only partially confirmed.

According to the literature, complete withdrawal from training or a significant reduction in training load can lead to performance decline and changes in anthropometric characteristics [25,26]. This phenomenon is known as detraining [24], and it is typically classified as short-term (<4 weeks) or long-term (>4 weeks). However, it should be noted that a short-term reduction in training volume, if accompanied by the maintenance of high intensity, can enhance performance through a process called tapering [27,28]. Of course, the transitional period examined in this study was much longer, and the observed training reduction corresponds to detraining rather than tapering.

As previously mentioned, the off-season period—free from competitive obligations—offers an excellent opportunity for the physical development of football players, especially during adolescence when the potential for improvement is significant. This premise formed the basis of the present study, which investigated the effects of a transitional training program on key physical performance indicators.

Regarding body composition, the literature presents conflicting findings on the impact of detraining, particularly concerning body weight and body fat percentage. In the present study, no changes were observed in the body composition of the young football players. Some studies have reported no significant changes after short periods of detraining [29,30,31,32], while others have observed increases in both body weight and fat percentage [33,34]. These discrepancies may be attributed to factors such as daily caloric intake, the duration and extent of training cessation or reduction, the training status of the athletes, and their baseline anthropometric characteristics [11,35]. Moreover, lifestyle changes during this period (e.g., increased free time, more frequent social gatherings) [35] may influence anthropometric parameters even when some training is maintained.

Previous studies have consistently shown a decline in aerobic performance following a break from training [26,29,30,32,36,37]. For example, Koundourakis et al. [29] reported a reduction in VO_2_ max among professional footballers after a six-week transitional period, during which the first two weeks involved complete rest, and the remaining four weeks included low-intensity aerobic running (50–60% VO_2_ max) for 20–30 min, three times per week. In another study [30], semi-professional players who maintained high-intensity training during a five-week transition period preserved their performance in the YoYo Intermittent Recovery Test Level 2 (YYIR2). In contrast, a group that ceased training for two weeks showed a significant performance decline in YYIR2, which was reversed after two weeks of structured training. Similarly, in a recent study [26], young players who refrained from training for three weeks demonstrated reduced performance in the YoYoIRT1, regardless of whether they belonged to the control, plyometric, or combined HIIT + plyometric training groups. Only the group that performed high-intensity interval training (HIIT) alone maintained their performance.

In the current study, although no intermediate measurements were taken after the initial two weeks of rest, a significant increase in VO_2_ max was observed compared to the pre-intervention baseline, following the subsequent four-week training program. Notably, in contrast to some prior studies where only maintenance of aerobic capacity was reported, the present study demonstrated actual improvement. This discrepancy could be attributed to differences in intervention program design (e.g., duration, frequency, intensity) and athlete training status, as more highly trained individuals may be more susceptible to detraining effects [38]. Moreover, while this study and the work of Koundourakis et al. [29] used VO_2_ max to assess aerobic capacity, other studies used different variations of the YoYo test.

No changes were observed in any of the isokinetic torque parameters after the transitional period, except at 60°/s in the knee extensors of the right leg, where a decrease in performance was recorded. Specifically, the mean value decreased from 238 Nm to 225 Nm. This finding was unexpected and cannot be explained, as no similar change was observed in any other isokinetic torque indicator. Our findings regarding isokinetic torque are in agreement with several other studies that also reported no significant changes. Previous studies examining short-term detraining periods have generally reported no significant changes in muscular strength [25,39,40,41,42]. Similarly, studies incorporating transitional programs have yielded comparable results. For instance, in the aforementioned study by Joo (2018) [30], no changes were observed in lower-limb isokinetic torque at any movement velocity (60°/s, 180°/s, 240°/s) across intervention groups. Another study in young football players also found no changes after 4 weeks of rest followed by 4 weeks of training. However, Chatzinikolaou et al. (2018) [31] observed a significant reduction in isokinetic strength in a control group after five weeks without training. Conversely, the intervention group not only maintained but improved its strength levels. It is worth noting that this study involved early pubertal athletes (ages 13–15, Tanner Stage 3) and that the effects of detraining depend on factors such as biological maturation, training history, and training load [43].

In the present study, it was observed that performance in the CMJ declined after the transitional period, whereas no such decrease was noted in the SJ. In the study by Liou [26], the control group showed performance declines, the HIIT groups maintained performance, and the plyometric group improved. These findings emphasize the importance of training specificity. The transitional program in the current study appeared to have minimal effect on the stretch-shortening cycle, which is a key determinant of CMJ performance [44,45]. In two studies by Koundourakis et al. [29,32], reductions in both CMJ and SJ performance were reported among professional players, and similar results were found for the control group in the adolescent study by Chatzinikolaou [31]. However, that study also showed that a properly designed strength program could improve jump performance in youth players.

This study has some limitations. First, the absence of a control group limited the ability to quantify the specific effect of the intervention. A measurement after the two-week detraining phase would have provided a clearer picture of performance decline prior to program initiation. Additionally, including more performance indicators (e.g., speed, agility, repeated sprint ability) would have contributed to a more comprehensive understanding of physical fitness adaptations in this age group.

## 5. Conclusions

The transition period can be strategically utilized by coaching staff working with youth football players to improve selected physical abilities. Specifically, after a two-week break from training—which is essential for both physical and especially psychological recovery—the implementation of a structured five-day-per-week program focusing on aerobic capacity and strength may lead to improvements in VO_2_ max and maintenance of isokinetic strength, power output, and body composition.

## Figures and Tables

**Table 1:** Descriptive Statistics and Paired-Samples *t*-test Results for Pre- and Post-Intervention Anthropometric and Physiological Measures.

Variable	Pre	Post				
	Mean ± SD (95% CI)	Mean ± SD (95% CI)	t	*p*	Cohen’s d	Cohen’s d 95% CI
Height (m)	1.78 ± 0.07 (1.74–1.83)	1.79 ± 0.07 (1.74–1.83)	−1.443	0.175	−0.400	−0.959–0.173
Body weight (kg)	70.3 ± 8.4 (65.2–75.4)	70.3 ± 7.6 (65.7–74.9)	0.001	0.999	0.001	−0.544–0.544
BMI	22.0 ± 1.3 (21.3–22.8)	22.0 ± 1.0 (21.3–22.6)	0.546	0.595	0.151	−0.399–0.695
Body Fat (%)	8.1 ± 2.7 (6.5–9.8)	8.2 ± 2.9 (6.4–9.9)	−0.024	0.981	−0.007	−0.550–0.537
Muscle mass (kg)	61.5 ± 6.2 (57.7–65.2)	61.3 ± 5.7 (57.8–64.8)	0.412	0.688	0.114	−0.434–0.657
Fat mass (kg)	5.5 ± 2.6 (4.0–7.1)	5.8 ± 2.6 (4.2–7.4)	−0.941	0.365	−0.261	−0.809–0.297

**Table 2:** Descriptive Statistics and Paired-Samples *t*-test Results for Cardiovascular and Cardiorespiratory Variables Between Pre- and Post-Intervention Assessments.

Variable	Pre	Post				
	Mean ± SD (95% CI)	Mean ± SD (95% CI)	t	*p*	Cohen’s d	Cohen’s d 95% CI
Resting Heart Rate (bpm)	65 ± 12 (58–73)	67 ± 10 (61–73)	−0.553	0.590	−0.153	−0.697–0.397
Systolic (mmHg)	122 ± 10 (116–128)	120 ± 8 (115–125)	0.472	0.646	0.131	−0.418–0.674
Diastolic (mmHg)	64 ± 7 (59–68)	63 ± 6 (59–67)	0.301	0.768	0.084	−0.463–0.626
Anaerobic Threshold Time (s)	441 ± 24 (426–455)	448 ± 20 (436–460)	−1.589	0.138	−0.441	−1.003–0.139
Anaerobic Threshold HR (bpm)	180 ± 7 (176–184)	179 ± 6 (176–183)	0.439	0.668	0.122	−0.426–0.665
Anaerobic Threshold Velocity (km/h)	14.9 ± 0.9 (14.4–15.4)	15.2 ± 0.7 (14.7–15.6)	−1.620	0.131	−0.449	−1.013–0.131
Anaerobic Threshold VO_2_ max (mL/kg/min)	50.0 ± 4.9 (47.0–53.0)	51.1 ± 4.6 (48.4–54.0)	−1.132	0.280	−0.314	−0.865–0.250
Maximal Exercise Time (s)	539 ± 30 (521–557)	542 ± 23 (529–556)	−0.619	0.547	−0.172	−0.716–0.380
Maximal Heart Rate (bpm)	195 ± 9 (189–200)	195 ± 7 (191–200)	−0.361	0.724	−0.100	−0.643–0.447
Maximal Velocity (km/h)	18.2 ± 1.0 (17.6–18.8)	18.3 ± 0.8 (17.8–18.8)	−0.433	0.673	−0.120	−0.663–0.428
VO_2_ max (mL/kg/min)	55.4 ± 5.1 (52.3–58.4)	57.1 ± 4.6 (54.3–59.9)	−2.348	0.037 *	−0.651	−1.242–(−0.039)
VO_2_ max (mL/min)	3871 ± 539 (3545–4197)	4022 ± 407 (3776–4268)	−2.313	0.039 *	−0.641	−1.231–(−0.031)
Respiratory Exchange Ratio (RER)	1.16 ± 0.03 (1.14–1.18)	1.17 ± 0.03 (1.15–1.19)	−1.190	0.257	−0.330	−0.883–0.235

Note: * *p* < 0.05.

**Table 3:** Descriptive Statistics and Paired-Samples *t*-test Results for Isokinetic Strength and Jump Performance Between Pre- and Post-Intervention Assessments.

Variable	Pre			Post						
	Mean ± SD (95% CI)			Mean ± SD (95% CI)			t	*p*	Cohen’s d	Cohen’s d 95% CI
Right Knee Extensors 60°/s (Nm)	238.1 ± 34.2	217.5	258.8	224.6 ± 35.7	203.1	246.2	2.299	0.040 *	0.638	0.027–1.226
Right Knee Extensors 180°/s (Nm)	171.9 ± 22.7	158.2	185.6	174.7 ± 22.1	161.3	188.1	−0.976	0.348	−0.271	−0.819–0.289
Right Knee Extensors 300°/s (Nm)	132.7 ± 17.2	122.3	143.1	135.5 ± 19.3	123.8	147.1	−1.235	0.240	−0.343	−0.896–0.224
Right Knee Flexors 60°/s (Nm)	154.7 ± 29.8	136.7	172.7	149.2 ± 24.1	134.7	163.8	1.480	0.165	0.411	−0.165–0.970
Right Knee Flexors 180°/s (Nm)	117.9 ± 16.3	108.0	127.7	118.0 ± 15.6	108.6	127.4	−0.043	0.966	−0.012	−0.555–0.532
Right Knee Flexors 300°/s (Nm)	95.1 ± 15.5	85.7	104.4	96.0 ± 17.1	85.7	106.3	−0.331	0.747	−0.092	−0.635–0.455
Left Knee Extensors 60°/s (Nm)	211.5 ± 42.5	185.8	237.2	212.7 ± 39.3	189.0	236.4	−0.265	0.795	−0.074	−0.616–0.472
Left Knee Extensors 180°/s (Nm)	165.7 ± 29.3	148.0	183.4	162.3 ± 24.6	147.4	177.2	1.393	0.189	0.386	−0.186–0.944
Left Knee Extensors 300°/s (Nm)	127.5 ± 21.4	114.6	140.4	124.5 ± 18.6	113.2	135.7	1.327	0.209	0.368	−0.202–0.924
Left Knee Flexors 60°/s (Nm)	140.7 ± 25.5	125.3	156.1	139.2 ± 30.4	120.8	157.5	0.259	0.800	0.072	−0.474–0.615
Left Knee Flexors 180°/s (Nm)	116.9 ± 17.2	106.4	127.3	113.1 ± 17.6	102.5	123.7	1.211	0.249	0.336	−0.230–0.889
Left Knee Flexors 300°/s (Nm)	93.8 ± 13.7	85.5	102.0	93.2 ± 19.6	81.3	105.0	0.184	0.857	0.051	−0.494–0.594
CMJ (cm)	41.4 ± 5.8	37.9	44.9	39.2 ± 6.9	35.0	43.3	3.112	0.009 *	0.863	0.208–1.492
SJ (cm)	38.9 ± 6.5	35.0	42.8	37.2 ± 6.4	33.3	41.1	1.927	0.078	0.534	−0.058–1.108

Note: * *p* < 0.05.

## Data Availability

The original contributions presented in the study are included in the article, further inquiries can be directed to the corresponding author/s.

## References

[1] FIFA (2021). Professional Football. https://publications.fifa.com/en/annual-report-2021/around-fifa/professional-football-2021/.

[2] FIFA Advancing Football. https://inside.fifa.com/advancing-football.

[3] Sarmento H., Anguera M.T., Pereira A., Araújo D. (2018). Talent Identification and Development in Male Football: A Systematic Review. Sports Med..

[4] Vardakis L., Michailidis Y., Topalidis P., Zelenitsas C., Mandroukas A., Gissis I., Christoulas K., Mavrommatis G., Metaxas T. (2023). Application of a Structured Training Plan on Different-Length Microcycles in Soccer—Internal and External Load Analysis between Training Weeks and Games. Appl. Sci..

[5] Brito de Souza D., López-Del Campo R., Blanco-Pita H., Resta R., Del Coso J. (2019). An Extensive Comparative Analysis of Successful and Unsuccessful Football Teams in LaLiga. Front. Psychol..

[6] Plakias S., Michailidis Y. (2024). Factors Affecting the Running Performance of Soccer Teams in the Turkish Super League. Sports.

[7] Barca Innovation Hub The Increase in Football Matches Heightens the Risk of Devaluation and Loss of Product Quality. https://barcainnovationhub.fcbarcelona.com/blog/the-increase-in-football-matches-heightens-the-risk-of-devaluation-and-loss-of-product-quality/.

[8] Morgans R., Orme P., Anderson L., Drust B. (2014). Principles and Practices of Training for Soccer. J. Sport Health Sci..

[9] Silva J.R., Brito J., Akenhead R., Nassis G.P. (2016). The Transition Period in Soccer: A Window of Opportunity. Sports Med..

[10] Vassilis S., Yiannis M., Athanasios M., Dimitrios M., Ioannis G., Thomas M. (2019). Effect of a 4-Week Detraining Period Followed by a 4-Week Strength Program on Isokinetic Strength in Elite Youth Soccer Players. J. Exerc. Rehabil..

[11] Clemente F.M., Ramirez-Campillo R., Sarmento H. (2021). Detrimental Effects of the Off-Season in Soccer Players: A Systematic Review and Meta-Analysis. Sports Med..

[12] Padrón-Cabo A., Lorenzo-Martínez M., De Dios-Álvarez V., Rey E., Solleiro-Durán D. (2025). Effects of a Short-Term Detraining Period on the Physical Fitness in Elite Youth Soccer Players: A Comparison between Chronological Age Groups. J. Strength Cond. Res..

[13] Clemente F.M., Soylu Y., Arslan E., Kilit B., Garrett J., van den Hoek D., Badicu G., Silva A.F. (2022). Can High-Intensity Interval Training and Small-Sided Games Be Effective for Improving Physical Fitness after Detraining? A Parallel Study Design in Youth Male Soccer Players. PeerJ.

[14] McKay A.K.A., Stellingwerff T., Smith E.S., Martin D.T., Mujika I., Goosey-Tolfrey V.L., Sheppard J., Burke L.M. (2022). Defining Training and Performance Caliber: A Participant Classification Framework. Int. J. Sports Physiol. Perform..

[15] Mandroukas A., Michailidis Y., Metaxas T. (2023). Muscle Strength and Hamstrings to Quadriceps Ratio in Young Soccer Players: A Cross-Sectional Study. J. Funct. Morphol. Kinesiol..

[16] Brink-Elfegoun T., Kaijser L., Gustafsson T., Ekblom B. (2007). Maximal Oxygen Uptake Is Not Limited by Central Nervous System Governor. J. Appl. Physiol..

[17] Åstrand P.O., Rodahl K. (1986). Evaluation of Physical Performance on the Basis of Tests. Textbook of Work Physiology.

[18] Freitas T.T., Pereira L.A., Alcaraz P.E., Arruda A.F., Guerriero A., Azevedo P.H., Loturco I. (2019). Influence of Strength and Power Capacity on Change of Direction Speed and Deficit in Elite Team-Sport Athletes. J. Hum. Kinet..

[19] Faul F., Erdfelder E., Buchner A., Lang A.G. (2009). Statistical Power Analyses Using G*Power 3.1: Tests for Correlation and Regression Analyses. Behav. Res. Methods.

[20] Faul F., Erdfelder E., Lang A.G., Buchner A. (2007). G*Power 3: A Flexible Statistical Power Analysis Program for the Social, Behavioral, and Biomedical Sciences. Behav. Res. Methods.

[21] The Jamovi Project (2025). Jamovi.

[22] IBM Corporation (2025). IBM SPSS Statistics for Windows.

[23] JASP Team (2025). JASP.

[24] Cohen J. (1988). Statistical Power Analysis for the Behavioral Sciences.

[25] Mujika I., Padilla S. (2000). Detraining: Loss of Training-Induced Physiological and Performance Adaptations. Part I. Short-Term Insufficient Training Stimulus. Sports Med..

[26] Liu G., Wang X., Xu Q. (2024). Supervised Offseason Training Programs Are Able to Mitigate the Effects of Detraining in Youth Men Soccer Players Physical Fitness: A Randomized Parallel Controlled Study. J. Sports Sci. Med..

[27] Aubry A., Hausswirth C., Louis J., Coutts A.J., Le Meur Y. (2014). Functional Overreaching: The Key to Peak Performance during the Taper?. Med. Sci. Sports Exerc..

[28] Mujika I., Padilla S. (2003). Scientific Bases for Precompetition Tapering Strategies. Med. Sci. Sports Exerc..

[29] Koundourakis N.E., Androulakis N., Dermitzaki E., Venihaki M., Margioris A.N. (2019). Effect of a 6-Week Supervised Detraining Period on Bone Metabolism Markers and Their Association with Ergometrics and Components of the Hypothalamic-Pituitary-Gonadal (HPG) Axis in Professional Male Soccer Players. J. Bone Miner. Metab..

[30] Joo C.H. (2018). The Effects of Short-Term Detraining and Retraining on Physical Fitness in Elite Soccer Players. PLoS ONE.

[31] Chatzinikolaou A., Michaloglou K., Avloniti A., Leontsini D., Deli C.K., Vlachopoulos D., Gracia-Marco L., Arsenis S., Athanailidis I., Draganidis D. (2018). The Trainability of Adolescent Soccer Players to Brief Periodized Complex Training. Int. J. Sports Physiol. Perform..

[32] Koundourakis N.E., Androulakis N.E., Malliaraki N., Tsatsanis C., Venihaki M., Margioris A.N. (2014). Discrepancy between Exercise Performance, Body Composition, and Sex Steroid Response after a Six-Week Detraining Period in Professional Soccer Players. PLoS ONE.

[33] Suarez-Arrones L., Lara-Lopez P., Maldonado R., Torreno N., De Hoyo M., Nakamura F.Y., Di Salvo V., Mendez-Villanueva A. (2019). The Effects of Detraining and Retraining Periods on Fat-Mass and Fat-Free Mass in Elite Male Soccer Players. PeerJ.

[34] Sotiropoulos A., Travlos A.K., Gissis I., Souglis A.G., Grezios A. (2009). The Effect of a 4-Week Training Regimen on Body Fat and Aerobic Capacity of Professional Soccer Players during the Transition Period. J. Strength Cond. Res..

[35] Rossi F.E., Landreth A., Beam S., Jones T., Norton L., Cholewa J.M. (2017). The Effects of a Sports Nutrition Education Intervention on Nutritional Status, Sport Nutrition Knowledge, Body Composition, and Performance during Off-Season Training in NCAA Division I Baseball Players. J. Sports Sci. Med..

[36] Thomassen M., Christensen P.M., Gunnarsson T.P., Nybo L., Bangsbo J. (2010). Effect of Two-Week Intensified Training and Inactivity on Muscle Na^+^-K^+^ Pump Expression, Phospholemman (FXYD1) Phosphorylation, and Performance in Soccer Players. J. Appl. Physiol..

[37] Christensen P.M., Krustrup P., Gunnarsson T.P., Kiilerich K., Nybo L., Bangsbo J. (2011). VO_2_ Kinetics and Performance in Soccer Players after Intense Training and Inactivity. Med. Sci. Sports Exerc..

[38] Coyle E.F., Martin W.H., Sinacore D.R., Joyner M.J., Hagberg J.M., Holloszy J.O. (1984). Time Course of Loss of Adaptations after Stopping Prolonged Intense Endurance Training. J. Appl. Physiol..

[39] Chaouachi A., Ben Othman A., Makhlouf I., Young J.D., Granacher U., Behm D.G. (2019). Global Training Effects of Trained and Untrained Muscles with Youth Can Be Maintained during Four Weeks of Detraining. J. Strength Cond. Res..

[40] Joo C.H. (2016). The Effects of Short-Term Detraining on Exercise Performance in Soccer Players. J. Exerc. Rehabil..

[41] Lehnert M., Psotta R., Chvojka P., Ste Croix M.D. (2014). Seasonal Variation in Isokinetic Peak Torque in Youth Soccer Players. Kinesiology.

[42] Mujika I., Padilla S. (2000). Detraining: Loss of Training-Induced Physiological and Performance Adaptations. Part II: Long-Term Insufficient Training Stimulus. Sports Med..

[43] Faigenbaum A.D., Kraemer W.J., Blimkie C.J., Jeffreys I., Micheli L.J., Nitka M., Rowland T.W. (2009). Youth Resistance Training: Updated Position Statement Paper from the National Strength and Conditioning Association. J. Strength Cond. Res..

[44] Marković G., Jukić I., Milanović D., Metikoš D. (2007). Effects of Sprint and Plyometric Training on Muscle Function and Athletic Performance. J. Strength Cond. Res..

[45] Marković G., Mikulić P. (2010). Neuro-Musculoskeletal and Performance Adaptations to Lower-Extremity Plyometric Training. Sports Med..

